# Retraction: Enhanced adsorption of ionizable aromatic compounds on humic acid-coated carbonaceous adsorbents

**DOI:** 10.1039/d0ra90039g

**Published:** 2020-04-23

**Authors:** Yubing Sun, Changlun Chen, Dadong Shao, Jiaxing Li, Xiaoli Tan, Guixia Zhao, Shubin Yang, Xiangke Wang

**Affiliations:** Key Laboratory of Novel Thin Film Solar Cells, Institute of Plasma Physics, Chinese Academy of Sciences P.O. Box 1126 Hefei 230031 P. R. China xkwang@ipp.ac.cn +86 5515591310 +86 551 5592788

## Abstract

Retraction of ‘Enhanced adsorption of ionizable aromatic compounds on humic acid-coated carbonaceous adsorbents’ by Yubing Sun *et al.*, *RSC Adv.*, 2012, **2**, 10359–10364, DOI: 10.1039/C2RA21713A.

The Royal Society of Chemistry, with the agreement of the named authors, hereby wholly retracts this *RSC Advances* article due to concerns with the reliability of the data in the published article.

Repeating fragments can be observed in the XRD spectrum for GONs in Fig. 1C, which indicates that it has been manipulated. In addition, there are unexpected similarities in the baseline of the XRD data presented for MWCNTs and AC (Fig. 1C) in the 32.5–40° and 45–57.5° regions.

Given the significance of the concern about the validity of the data, the findings presented in this paper are no longer reliable.

Signed: Yubing Sun, Dadong Shao, Guixia Zhao, Shubin Yang and Xiangke Wang

Date: 27^th^ March 2020

Changlun Chen, Jiaxing Li and Xiaoli Tan were contacted but did not respond.

Retraction endorsed by Laura Fisher, Executive Editor, *RSC Advances*

## Supplementary Material

